# Exploring Multipartite Steering Effect Using Bell Operators

**DOI:** 10.3390/e22010019

**Published:** 2019-12-23

**Authors:** Li-Yi Hsu, Shoichi Kawamoto

**Affiliations:** Department of Physics, Chung Yuan Christian University, Chungli 32081, Taiwan; kawamoto@cycu.edu.tw

**Keywords:** quantum steering effect, Bell operators, quantum network

## Abstract

While Bell operators are exploited in detecting Bell nonlocality and entanglement classification, we demonstrate their usefulness in exploring Einstein–Podolsky–Rosen (EPR) steering, which represents the quantum correlation intermediate between entanglement and Bell nonlocality. We propose a task function that detects steerability of multi-qubit states in bipartite scenarios. A novel necessary and sufficient steering criterion is based on the superposition of the recursive Bell operators which are often employed for detecting Bell nonlocality. Utilizing the task function we can (i) reveal the one-to-one mapping relation between joint measurability and unsteerability, (ii) geometrically depict and compare the entanglement classification and the steering criteria and propose a geometrical measure, and (iii) compare the EPR steering with Bell nonlocality using an alternative task function. We extend the result to detect EPR steering for multi-qutrit cases and some numerical results are illustrated as examples. Finally, the steering criteria in a star-shaped quantum network is studied to see how the result is applied to a genuine multipartite steering case.

## 1. Introduction

Entanglement, steerability, and Bell nonlocality are three quantum aspects distinguishable from classical physics. Although they are not equivalent [1,2,3,4], they are closely inter-related. On the one hand, there is a hierarchal structure of three types of nonclassical correlation/nonlocality represented by these three aspects [2,5]. Bell nonlocality refers to the strongest type of quantum correlation that cannot be reproduced by using any local hidden variable (LHV) model. Entanglement is characterized by the inseparability of quantum composite systems; it cannot be modeled in terms of separable local states. Given any entangled state, there exists an entanglement witness to detect it [6]. An entanglement witness can demonstrate correlations that cannot be reproduced by using any combination of local hidden state (LHS) models. The notion of quantum steering was first introduced by Schrödinger [7] in response to the Einstein–Podolsky–Rosen (EPR) paradox [8]. When two systems are ‘entangled’, one party, through a local measurement on the accessible systems, can steer or pilot the state of the other remote system. EPR steering is an intermediate type of quantum correlation between Bell nonlocality and entanglement. The steering effect can be exploited to characterize the evident ability of nonlocality by [2,9,10,11], and witnessing steering implies entanglement certification [12]. In quantum information, the EPR-steering has attracted much attention since it provides an alternative advantage involving randomness extraction [13], subchannel discrimination [14], one-sided device-independent quantum key distribution [15], and quantum communication [16,17].

There are two operational definitions of quantum steering. Let a bipartite state be distributed by two spatially separated parties, Alice and Bob. To demonstrate steerability from Alice to Bob, Alice as the steering party performs her local measurements on her particles. Therein, Alice’s measurement devices are untrusted while Bob’s are trusted. This state is steerable if and only if the unnormalized conditional post-measured states in Bob’s hand, often referred to as a measurement assemblage, cannot be described by any combination of LHS and LHV models. Various steering criteria or inequalities based on this definition have been proposed [1,2,18,19,20,21].

In this paper, we employ the other definition for EPR steering as a task with two parties [2,17]. In this task, all measurement devices for both sides are trusted but Alice can be dishonest. Alice’s task is to convince Bob of her steerability on the qubits in Bob’s hand, and she is allowed to inform Bob of her local measurement settings and outcomes via one-way classical communication. If Alice is honest, she prepares an *n*-qubit entangled state *W* and sends (n−k) qubits from it to Bob. In contrast, if Alice is dishonest, she sets up an LHV λ with a probability $p_{\lambda }$, sends Bob local (n−k)-qubit pre-existing states with density matrices $\rho_{\lambda }$, and announces forged measurement settings and outcomes via classical communication. In this case, Bob’s assemblage of this state can be reproduced using the LHS model $\left\{\rho_{\lambda },p_{\lambda }\right\}$. A quantum state is unsteerable if and only if it can be simulated using a combination of LHV and LHS (denoted as LHV–LHS, or V–S for short) models; otherwise, it is steerable [1,2,22]. To verify Alice’s steering ability, Bob can evaluate the strength of the bipartite correlation using a task function based on his local operations and one-way classical communication. As a steering criteria, since quantum steering is a type of correlation stronger than that in terms of any LHV–LHS model, Bob confirms EPR steering if and only if the value of the task function is larger than the upper bound of the task function value using any LHV–LHS model. Therein, Bell operators play an essential role in bipartite correlation evaluation and hence steering witness.

In this paper, we explore a novel way of detecting multiqubit/multiqutrit steering with Bell operators; in the pioneer work [23,24], the two-level multipartite steering inequalities were proposed based on the Mermin-type inequalities [25] in which the proposed inequalities are analogous to Bell-type inequalities. We extend the ideas and results from two-qubit cases [26,27] to multi-qubit cases. Instead of directly characterizing the steerability of two non-commuting Bell operators, we evaluate the steerability of their superposition. On one hand, as a combination of the analogs of the Klyshko-type Bell inequalities and entanglement classification, the proposed nonlinear steering inequalities can reveal the one-to-one mapping relation between joint measurability and unsteerability. On the other hand, besides detecting Bell nonlocality, Bell operators are also employed for entanglement classification [28,29] and entanglement witness [30]. Therefore, through Bell operators, the proposed criteria can be regarded as resources for demonstrating EPR steering compared to Bell nonlocality and entanglement.

The remainder of this paper is organized as follows: In Section 2, we introduce the Bell operators appeared in the Klyshko-type inequalities, which can be regarded as the generalization of the Clauser–Horne–Shimony–Holt (CHSH) inequality [31,32,33,34]. Specifically, these Bell operators can be explicitly expressed as recursive forms, and are exploited in an entanglement classification. In Section 3, we propose novel nonlinear steering inequalities for the bipartition of *n*-qubit multipartite states. The connection between unsteerability and joint measurability is argued. We derive the necessary and sufficient criteria of unsteerability, and the related measure of steering is given from the geometrical viewpoint. The EPR steering in qutrit systems is considered in Section 4. The recursive Bell operators are also found to be useful for drawing nonlinear steering criteria, and some numerical results are presented. In Section 5, the steering effect in a star-shaped quantum network is analyzed; here, the source center tries to steer the states of many end-users. This is a type of genuine multipartite steering, and the steering inequality for this network is proposed. Finally, the conclusion is drawn in Section 6.

## 2. Preliminaries

Denote the *n*-qubit Klyshko-type Bell operators by

(1) $$\begin{array}{cc} B_{n} & =B_{k}^{+}B_{n-k}+B_{k}^{-}B_{n-k}^{\prime } \end{array}$$

(2) $$\begin{array}{cc} \; & =B_{k}B_{n-k}^{+}+B_{k}^{\prime }B_{n-k}^{-}, \end{array}$$

(3) $$\begin{array}{cc} B_{n}^{\prime } & =B_{k}^{+}B_{n-k}^{\prime }-B_{k}^{-}B_{n-k} \end{array}$$

(4) $$\begin{array}{cc} \; & =B_{k}^{\prime }B_{n-k}^{+}-B_{k}B_{n-k}^{-}, \end{array}$$

where $B_{k}^{\pm }=\frac{1}{2}\left(B_{k}\pm B_{k}^{\prime }\right)$ and 1≤k≤n−1 [35,36]. The one-qubit Bell operators $B_{1}$ and $B_{1}^{\prime }$ on the *j-*th subsystem are spin observables $M_{j}^{0}=\vec{u_{j}}\cdot \vec{\sigma }$ and $M_{j}^{1}=\vec{v_{j}}\cdot$$\vec{\sigma }$, respectively, where $\vec{\sigma }=\left(\sigma_{x},\sigma_{y},\sigma_{z}\right)$ is a vector of Pauli matrices, and $\vec{u_{j}}\;$ and $\vec{v_{j}}$ both are the unit vectors on the Bloch sphere. These operators can also be recursively obtained from the fundamental relation, $B_{n}=\frac{1}{2}\left[B_{n-1}\left(M_{n}^{0}+M_{n}^{1}\right)+B_{n-1}^{\prime }\left(M_{n}^{0}-M_{n}^{1}\right)\right]$ and the other one by exchanging all the non-primed $B_{k}$ and primed $B_{k}^{\prime }$. For example, the two-qubit Bell operator reads

$$B_{2}=\frac{1}{2}\left[M_{1}^{0}M_{2}^{0}+M_{1}^{1}M_{2}^{0}+M_{1}^{0}M_{2}^{1}-M_{1}^{1}M_{2}^{1}\right]\;,$$

which is nothing but the CHSH operator multiplied with the extra factor $\frac{1}{2}$. The Bell operator $B_{n}^{\prime }$ is given by the same expression $B_{n}$ but with $M_{j}^{0}$↔$M_{j}^{1}$ for each *j*. In the following, we denote the outcomes of $M_{j}^{i}$ by $o_{j}^{i}$, where i∈{0,1} and $o_{j}^{i}$∈{1,−1}. Notably, the Bell operators $B_{n}$ and $B_{n}^{\prime }$ can be exploited in the entanglement classification as follows [28,29]. Given the *n*-qubit state, we have

(5) $$\sqrt{{\langle B_{n}\rangle }^{2}+{\langle B_{n}^{\prime }\rangle }^{2}}\leq \sqrt{2^{E_{n}-1}},$$

where the entanglement index of the *n*-qubit system is $E_{n}=n-K_{n}-2L_{n}+2$, $K_{n}$ is the number of separated single qubits, and $L_{n}$ is the number of groups into which the entangled $n-K_{n}$ qubits are divided with each group of qubits being fully entangled. Notably, RHS of (5) is different from (12) in [28] since the different two-qubit Bell operators are exploited.

Note that the upper bound appeared in RHS of (5) is tight and state-dependent. As a preparation for proposing steering criteria in the following sections, we look for a state-independent bound of *m*-qubit system which is given as a local hidden state. We follow Roy’s work to verify that the maximum of $E_{m}$ is *m* with $K_{m}=0$ and $L_{m}=1$, which can be achieved using the *m*-qubit Greenberger–Horne–Zeilinger (GHZ) state [37,38]. (In the next section, we apply the following result for the cases with *m* being n−k or $n^{\prime }-k^{\prime }$.) Firstly we introduce the Mermin-type Bell operators

(6) $$M_{m}^{+}=\frac{1}{2}\left({⊗}_{j=1}^{m}\sigma_{j}^{+}+{⊗}_{j=1}^{m}\sigma_{j}^{-}\right),\;M_{m}^{-}=\frac{1}{2i}\left({⊗}_{j=1}^{m}\sigma_{j}^{+}-{⊗}_{j=1}^{m}\sigma_{j}^{-}\right),$$

where $\sigma_{j}^{\pm }=\sigma_{x}\pm i\sigma_{y}$, and $\sigma_{x}$ and $\sigma_{y}$ are the usual Pauli operators. Without loss of generality, let $M_{j}^{1}$ be $\sigma_{y}$ and $M_{j}^{0}$ be either $\sigma_{x}$ or $-\sigma_{x}$ hereafter. Up to the phase −1 and for even *m*, we have either

$$B_{m}=\frac{1}{2^{\frac{m}{2}}}\left(M_{m}^{+}\pm M_{m}^{-}\right),\;B_{m}^{\prime }=\frac{1}{2^{\frac{m}{2}}}\left(M_{m}^{+}\mp M_{m}^{-}\right);$$

for odd *m*, we have either

$$B_{m}=\frac{1}{2^{\frac{m-1}{2}}}M_{m}^{\pm },\;B_{m}^{\prime }=\frac{1}{2^{\frac{m-1}{2}}}M_{m}^{\mp }.$$

For the *m*-qubit GHZ state $|\psi_{m}^{\theta }\rangle =\frac{1}{\sqrt{2}}\left({|0\rangle }^{⊗m}+e^{i\theta }{|1\rangle }^{⊗m}\right),$ we have [38]

(7) $$\langle \psi_{m}^{\theta }\left|M_{m}^{+}\right|\psi_{m}^{\theta }\rangle =2^{m-1}\cos\theta ,\;\langle \psi_{m}^{\theta }\left|M_{m}^{-}\right|\psi_{m}^{\theta }\rangle =2^{m-1}\sin\theta .$$

As a result, we reach a state-independent inequality

(8) $$\sqrt{{\langle B_{m}\rangle }^{2}+{\langle B_{m}^{\prime }\rangle }^{2}}\leq 2^{\frac{m-1}{2}}\equiv R_{m},$$

where the equality holds if the state is maximally-entangled with the corresponding index $E_{m}\;=\;m$ [23,24]. In this paper, we focus on a one-way steering effect, and for the (n−k)-qubit system (namely m=n−k), denote $R_{n-k}=2^{\frac{n-k-1}{2}}$ as the least upper bound of $\sqrt{{\langle B_{n-k}\rangle }^{2}+{\langle B_{n-k}^{\prime }\rangle }^{2}}$ in the quantum region, which can be achieved using the (n−k)-qubit GHZ state $|\psi_{n-k}^{\theta }\rangle \;=\;\frac{1}{\sqrt{2}}\left({|0\rangle }^{⊗n-k}+e^{i\theta }{|1\rangle }^{⊗n-k}\right)$.

## 3. Multipartite Criteria of Unsteerability

To test the Alice’s ability of steering Bob’s qubits, we consider the following bipartite communication task. Given a generic *n*-qubit state *W* distributed between Alice (*k*-qubit) and Bob ((n−k)-qubit), the goal is to maximize the value of a task function $F^{\left(n,k\right)}\left(W\right)$, where

(9) $$F^{\left(n,k\right)}\left(W\right)=\sqrt{{\langle B_{k}^{+}B_{n-k}\rangle }^{2}+{\langle B_{k}^{+}B_{n-k}^{\prime }\rangle }^{2}}+\sqrt{{\langle B_{k}^{-}B_{n-k}\rangle }^{2}+{\langle B_{k}^{-}B_{n-k}^{\prime }\rangle }^{2}}.$$

Notably, the first and second terms in the RHS are to quantify the steering ability of two observables, $B_{k}^{+}$ and $B_{k}^{-}$, which are the superpositions of the non-commuting Bell operators, $B_{k}$ and $B_{k}^{{}^{\prime }}$. Some remarks are made before proceeding further. Firstly, $F^{\left(2,1\right)}\left(W\right)$ has been extensively studied in [26,27], which is regarded as an analog of CHSH inequality for steering [27]. Later we will investigate the relation between steering and either nonlocality or entanglement classification using $F^{\left(n,k\right)}\left(W\right)$ and its generalized form. Secondly, it will be shown that the superposition of $B_{k}$ and $B_{k}^{{}^{\prime }}$ and the nonlinearity in (9) are necessary to reveal the strong connection between unsteerability and joint measurability [39,40]. Finally, as for the physical realization, the local observable measurements for testing EPR steering therein can also be exploited for testing nonlocality and entanglement classification.

In the protocol of this task, an honest Alice initially prepares an *n*-qubit state *W*. She keeps *k* qubits and sends the other (n−k) qubits to a distant Bob. Alice measures local observables, and then sends Bob the content of an input-output set $c=\{\left(M_{j}^{i_{j}},o_{j}^{i_{j}}\right)|1\leq j\leq k,$ $i_{j}\in \left\{0,1\right\}\}$ via one-way classical communication. On receiving *c*, Bob measures the observable either $M_{j^{\prime }}^{0}$ or $M_{j^{\prime }}^{1}$ on the $j^{\prime }$-th qubit at hand $\left(k+1\leq j^{\prime }\leq n\right)$. At last, Bob evaluates the value of $F^{\left(n,k\right)}\left(W\right)$ based on his local operations and one-way classical communication. Provided that the unsteerable state *W* is prepared by a dishonest Alice, all joint probability distributions can be simulated using a LHV–LHS model. In details, given a local hidden variable λ, Alice sends Bob (n−k)-qubit local states $\rho_{\lambda }$. The conditional output joint probability with the unsteerable *W* can be simulated using $\left\{\lambda ,\rho_{\lambda }\right\}$ such that

(10) $$P{\left(a,b|A,B\right)}_{V-S}=\sum_{\lambda }P\left(\lambda \right)P\left(a|A,\lambda \right)P\left(b|B,\rho_{\lambda }\right),$$

where the local input sets $A=\{M_{1}^{i_{1}},\ldots ,M_{k}^{i_{k}}\}$ and $B=\{M_{k+1}^{i_{k+1}},\ldots ,M_{n}^{i_{n}}\}$; and the local output sets $a=\{o_{1}^{i_{1}},\ldots ,o_{k}^{i_{k}}\}$ and $b=\{o_{k+1}^{i_{k+1}},\ldots ,o_{n}^{i_{n}}\}$. Importantly, given some local hidden variable λ, we have [37]

(11) $$\left|{\langle B_{k}\rangle }_{\lambda }\right|,\left|{\langle B_{k}^{\prime }\rangle }_{\lambda }\right|\leq 1,\;$$

(12) $$\left|{\langle B_{k}^{+}\rangle }_{\lambda }\right|+\left|{\langle B_{k}^{-}\rangle }_{\lambda }\right|\leq \max\left\{\left|{\langle B_{k}\rangle }_{\lambda }\right|,\left|{\langle B_{k}^{\prime }\rangle }_{\lambda }\right|\right\}\leq 1,$$

and

(13) $${\langle B_{n}^{\prime }\rangle }_{\left\{\lambda ,\rho_{\lambda }\right\}},{\langle B_{n}\rangle }_{\left\{\lambda ,\rho_{\lambda }\right\}}\leq \max_{\rho_{\lambda }}\left\{{\langle B_{n-k}\rangle }_{\rho_{\lambda }},{\langle B_{n-k}^{\prime }\rangle }_{\rho_{\lambda }}\right\}.$$

In the multipartite LHV–LHS model (10), we have

(14) $$\begin{array}{cc} F^{\left(n,k\right)} & \leq \max_{\rho_{\lambda }}\sqrt{{\langle B_{n-k}\rangle }_{\rho_{\lambda }}^{2}+{\langle B_{n-k}^{\prime }\rangle }_{\rho_{\lambda }}^{2}}\sum_{\lambda^{\prime }}P\left(\lambda^{\prime }\right)\left(\left|{\langle B_{k}^{+}\rangle }_{\lambda^{\prime }}\right|+\left|{\langle B_{k}^{-}\rangle }_{\lambda^{\prime }}\right|\right)\\ \; & \leq \max_{\rho_{\lambda }}\sqrt{{\langle B_{n-k}\rangle }_{\rho_{\lambda }}^{2}+{\langle B_{n-k}^{\prime }\rangle }_{\rho_{\lambda }}^{2}}. \end{array}$$

Therefore, according to (8), it is sufficient that if the state *W* is unsteerable, it must satisfy the steering inequality

(15) $$F^{\left(n,k\right)}\leq R_{n-k}.$$

It is noteworthy that the value ${\langle B_{n-k}\rangle }^{2}+{\langle B_{n-k}^{\prime }\rangle }^{2}$ are exploited as the test of the separable–inseparable (n−k)-particle density operators [28], or as the witness of full (n−k)-partite entanglement [29]. Regarding of the RHS inequality (15) as the least upper bound of $\sqrt{{\langle B_{n-k}\rangle }_{\mathrm{LHS}}^{2}+{\langle B_{n-k}^{\prime }\rangle }_{\mathrm{LHS}}^{2}}$, the necessary and sufficient condition for *W* to be unsteerable is

(16) $$F^{\left(n^{\prime },k^{\prime }\right)}\leq R_{n^{\prime }-k^{\prime }},$$

for all possible $k^{\prime }$(≤k) and $n^{\prime }-k^{\prime }$(≤n−k) subsystems chosen in Alice and Bob’s laboratories, respectively. In this case, Alice cannot convince Bob of her steering ability on any qubit in his hand. In the end, the equality in (16) holds for some $k^{\prime }$ and $n^{\prime }-k^{\prime }$ if $\rho_{\lambda }=$$|\psi_{n^{\prime }-k^{\prime }}^{\theta }\rangle \langle \psi_{n^{\prime }-k^{\prime }}^{\theta }|$ irrespective to λ.

To verify that (16) is indeed a steering inequality, we resort to the connection between the steerability and joint measurability in the two-level case [39,40]. In particular, it is shown that, for any set of incompatible observables, one can find an entangled state with which resulting statistics violates a steering inequality [40]. In the simplest case, where n=2 and k=1, the spin observables $M_{1}^{1}=M_{2}^{1}=\sigma_{y}$, $M_{1}^{0}=M_{2}^{0}=\sigma_{x}$, and the Bell state $|\psi_{2}^{\theta }\rangle$ initially shared between Alice and Bob. Notably, the observables $M_{1}^{0}$ and $M_{1}^{1}$ are presumed most incompatible [41], and the maximal value of the task function $F^{\left(2,1\right)}$ in the quantum region is $\sqrt{2}$. Now Alice performs the joint measurement on her half of entangled qubits, the probability distribution can be exactly simulated using the LHV–LHS model (10). In details, we denote the unsharpened observables of Alice’s qubit $m_{1}^{0}=\lambda_{0}M_{1}^{0}=\lambda_{0}\sigma_{x}$ and $m_{1}^{1}=\lambda_{1}M_{1}^{1}=\lambda_{1}\sigma_{y}$, $0<\lambda_{0},\lambda_{1}\leq 1$. Regarding Alice’s unsharp measurements, the condition $F^{\left(2,1\right)}=\frac{1}{2}\sqrt{{\langle \left(m_{1}^{0}+m_{1}^{1}\right)M_{2}^{0}\rangle }^{2}+{\langle \left(m_{1}^{0}+m_{1}^{1}\right)M_{2}^{1}\rangle }^{2}}+\frac{1}{2}\sqrt{{\langle \left(m_{1}^{0}-m_{1}^{1}\right)M_{2}^{0}\rangle }^{2}+{\langle \left(m_{1}^{0}-m_{1}^{1}\right)M_{2}^{1}\rangle }^{2}}\leq 1$ must be satisfied. Given the correlations $\langle M_{1}^{0}M_{2}^{1}\rangle =\langle M_{1}^{1}M_{2}^{0}\rangle =\sin\theta ,$$\langle M_{1}^{0}M_{2}^{0}\rangle =-\langle M_{1}^{1}M_{2}^{1}\rangle =\cos\theta$, we have

(17) $$\;\lambda_{0}^{2}+\lambda_{1}^{2}\leq 1,$$

which is the exact criteria of joint measurability for the most incompatible observable [42]. To realize such joint measurement, let the joint observable be [43,44]

$$G\left(i,\;j\right)=\frac{1}{4}\left(I_{2}+{\vec{\lambda }}_{ij}\cdot \vec{\sigma }\right),\;$$

where ${\vec{\lambda }}_{ij}=\left(i\lambda_{0},j\lambda_{1},0\right)$, $\left|{\vec{\lambda }}_{ij}\right|\leq 1$, and i, j∈{−1,1}. Obviously, we have G(i,j)≥0 and $\sum_{i,j}G(i,$ $j)=I_{2}$, $m_{1}^{0}=\sum_{j}G(+,$$j)-\sum_{j}G(-,$j), and $m_{1}^{1}=\sum_{i}G\left(i,+\right)-\sum_{i}G\left(i,-\right)$. Inversely, the condition (17) suffices the $\left|{\vec{\lambda }}_{ij}\right|\leq 1$ and hence G(i,j)≥0. Hence Alice can exploit the joint observable $\left\{G(i,j)\right\}$ that satisfy the steering inequality $F^{\left(2,1\right)}\leq 1$. Inversely, given (17), it is easy to verify that inequality $F^{\left(2,1\right)}\leq 1$ holds. When a prepared state *W* is a fully entangled state and Alice’s joint measurement consists of the two most incompatible observables, a straightforward calculation shows

(18) $$F^{\left(n,1\right)}\leq R_{n-1}\Leftrightarrow \lambda_{0}^{2}+\lambda_{1}^{2}\leq 1.$$

That is, if $m_{1}^{0}$ and $m_{1}^{1}$ are not jointly measurable ($\lambda_{0}^{2}+\lambda_{1}^{2}>1$) and hence incompatible, one can always find a fully entangled state $|\psi_{n}^{\theta }\rangle$ such that the resulting statistics violates a steering inequality $F^{\left(n,1\right)}\leq R_{n-1}$.

To explain why characterizing the steerability of two non-commuting operators is worse than characterizing the steerability of their superposition, we consider another task function using the terms $B_{k}B_{n-k}^{\pm }$ and $B_{k}^{\prime }B_{n-k}^{\pm }$ which appeared in (2) and (4),

(19) $$G^{\left(n,k\right)}=\sum_{B=B_{k},B_{k}^{\prime }}\sqrt{{\langle B(B_{n-k}+B_{n-k}^{\prime })\rangle }^{2}+{\langle B(B_{n-k}-B_{n-k}^{\prime })\rangle }^{2}},$$

where the first and second terms are exploited to evaluate the steering effect of $B_{k}$ and $B_{k}^{\prime }$ on the n−k qubits on which the Bell operators $B_{n-k}^{+}\;$ and $B_{n-k}^{-}$ are performed. In the LHV–LHS model, $G^{\left(n,k\right)}\leq \sqrt{2}\max_{\rho_{\lambda }}\sqrt{{\langle B_{n-k}\rangle }_{\mathrm{LHS}}^{2}+{\langle B_{n-k}^{\prime }\rangle }_{\mathrm{LHS}}^{2}}$ . Regarding the simplest case with n=2 and k=1, Alice’s joint measurement and the steering equality $G^{\left(2,1\right)}\leq \sqrt{2}$ leads to the trivial linear inequality $\lambda_{0}+\lambda_{1}\leq 2$. Eventually, it is the superposition of $B_{1}$ and $B_{1}^{\prime }$ as well as the nonlinearity in (9) that brings the quadratic inequality (17), and hence (18). As a result, $F^{\left(n,k\right)}$ rather than $G^{\left(n,k\right)}$ indeed reveals the one-to-one mapping relation between the unsteerability and joint measurability of the two most incompatible observables [45].

However, $\rho_{\lambda }$ is unknown to Bob in practice. We study Bob’s post-processing to increasing the value of the task function as follows. Let a hidden variable be a deterministic input-output set, which denotes $\lambda =\{\left(M_{j}^{i_{j}},o_{j}^{i_{j}}\right)|v_{\lambda }\left(M_{j}^{i_{j}}\right)=o_{j}^{i_{j}},1\leq j\leq k,$$i_{j}=0,1\}$. In the *k*-th round test, the untrusted Alice prepares a hidden variable $\lambda^{\left(k\right)}$ and sends Bob $\rho_{\lambda^{\left(k\right)}}$. Then Alice communicates Bob her local inputs and outputs $c^{\left(k\right)}$ as a subset of $\lambda^{\left(k\right)}$. We denote the LHS with the hidden variable λ and its sampling by $\;\rho_{\lambda }^{\Lambda }=\frac{1}{N_{\lambda }}\sum_{k,c^{\left(k\right)}\subset \lambda }^{}$ $\rho_{\lambda^{\left(k\right)}}$ and $S(\rho_{\lambda }^{\Lambda })$, respectively, where the $N_{\lambda }=\sum_{k,c^{\left(k\right)}\subset \lambda }^{}1$. The achievable tight upper-bound is

(20) $$F^{\left(n^{\prime },k^{\prime }\right)}\left(W\right)\leq \max_{S(\rho_{\lambda }^{\Lambda })}\sqrt{{\langle B_{n^{\prime }-k^{\prime }}\rangle }_{S(\rho_{\lambda }^{\Lambda })}^{2}+{\langle B_{n^{\prime }-k^{\prime }}^{\prime }\rangle }_{S(\rho_{\lambda }^{\Lambda })}^{2}}\leq R_{n^{\prime }-k^{\prime }},$$

for all $k^{\prime }$ and $n^{\prime }-k^{\prime }$ subsystems in Alice and Bob’s laboratories, respectively. As a result, even though Bob can locally increase the task function value through post-processing, the inequality (16) must hold, which leads to our main result.

**Theorem** **1.** *: An n-qubit state W is unsteerable if and only if Alice’s local operations, classical communication, and Bob’s post-processing cannot make the task function $F^{\left(n^{\prime },k^{\prime }\right)}$ defined in (9) larger than $R_{n^{\prime }-k^{\prime }}=2^{\frac{n^{\prime }-k^{\prime }-1}{2}}$ for $\forall n^{\prime }\leq n,$$k^{\prime }\leq k$, where $k^{\prime }$ is the subsystem of Alice and $n^{\prime }-k^{\prime }$ is that of Bob.*

The proof can be stated as follows.

**Proof.**  If (20) holds, one can simulate the probability distribution using LHV–LHS model with {λ, $\rho_{\lambda }^{\Lambda }\}$. Inversely, since *W* is unsteerable and hence $\left|\langle B_{k^{\prime }}\rangle \right|$, $\left|\langle B_{k}\rangle \right|\leq 1$, (20) is automatically satisfied.

**Figure 1:**
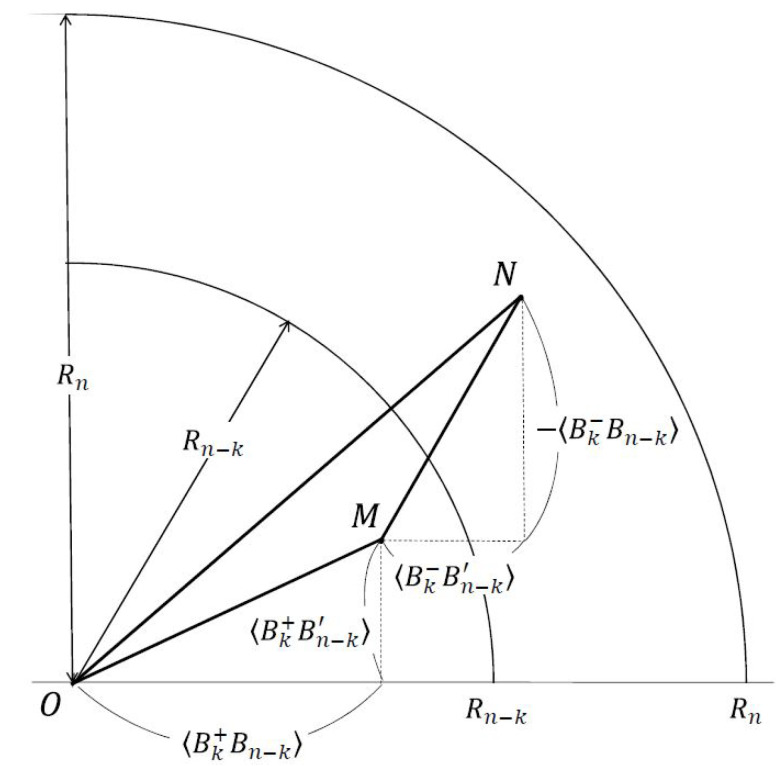
The geometry of $F_{2}^{\left(n,k\right)}$ and $T_{2}^{\left(n,k\right)}$. Without loss of generality, let $\pm {\langle B_{k}^{\pm }B_{n-k}\rangle }_{W}$ and ${\langle B_{k}^{\pm }B_{n-k}^{\prime }\rangle }_{W}$ all be positive for the steerable state *W*. $\vec{ON}=({\langle B_{n}\rangle }_{W}$, ${\langle B_{n}^{\prime }\rangle }_{W})$, $\vec{OM}=({\langle B_{k}^{+}B_{n-k}\rangle }_{W}$, ${\langle B_{k}^{+}B_{n-k}^{\prime }\rangle }_{W})$, and $\vec{MN}=({\langle B_{k}^{-}B_{n-k}^{\prime }\rangle }_{W}$, $-{\langle B_{k}^{-}B_{n-k}\rangle }_{W})$. By triangle inequality, $F_{2}^{\left(n,k\right)}\left(W\right)=\bar{OM}+\bar{MN}$$\geq \bar{ON}=T_{2}^{\left(n,k\right)}\left(W\right)$, where the equality holds if $\frac{{\langle B_{n}^{\prime }\rangle }_{W}}{{\langle B_{n}\rangle }_{W}}=\frac{{\langle B_{k}^{+}B_{n-k}^{\prime }\rangle }_{W}}{{\langle B_{k}^{+}B_{n-k}\rangle }_{W}}=-\frac{{\langle B_{k}^{+}B_{n-k}\rangle }_{W}}{{\langle B_{k}^{-}B_{n-k}^{\prime }\rangle }_{W}}$. The fact that $T_{2}^{\left(n,k\right)}\left(W\right)\geq R_{n-k}$ guarantees that $F_{2}^{\left(n,k\right)}\left(W\right)\geq R_{n-k}$ and hence the steerability of *W*. As for the measure, we have $S\left(W\right)=\frac{\bar{OM}+\bar{MN}-\bar{OP}}{R_{n}-R_{n-k}}\geq \frac{\bar{ON}-\bar{OP}}{R_{n}-R_{n-k}}=\frac{\bar{PN}}{R_{n}-R_{n-k}}$.

Specifically, regarding the $k^{\prime }$ systems at Alice’s side, (11) leads to $\sqrt{{\langle B_{k^{\prime }}\rangle }_{\lambda }^{2}+{\langle B_{k^{\prime }}^{\prime }\rangle }_{\lambda }^{2}}\leq \sqrt{2}$, where one equivalently sets $K_{k^{\prime }}=k^{\prime }$, $L_{k^{\prime }}=0$, and hence the entanglement index $E_{k^{\prime }}=2$. As a result, for the above LHV–LHS models, the achievable entanglement index is at most ($n^{\prime }-k^{\prime }$) since only ($n^{\prime }-k^{\prime }$) qubits are initially prepared. As an example, the equality $E_{n^{\prime }}=n^{\prime }-k^{\prime }$ with $K_{n^{\prime }}=k^{\prime }$ and $L_{n^{\prime }}=1$ holds if the local hidden state $\rho_{\lambda }=|\psi_{n^{\prime }-k^{\prime }}^{\theta }\rangle \langle \psi_{n^{\prime }-k^{\prime }}^{\theta }|$ and the local hidden variables $o_{j}^{i_{j}}=1$∀λ and $j\leq k^{\prime }$. Furthermore, from the geometrical viewpoint shown in Figure 1, we have

$$\sqrt{{\langle B_{n^{\prime }}\rangle }^{2}+{\langle B_{n^{\prime }}^{\prime }\rangle }^{2}}\leq F^{{}^{\left(n^{\prime },k^{\prime }\right)}}.$$

As a result, (20), $F^{\left(n^{\prime },\;k^{\prime }\right)}\leq R_{n^{\prime }-k^{\prime }}$ and (5) indeed indicate that entanglement index of the $n^{\prime }$-system must not be larger than $n^{\prime }-k^{\prime }$. Since there is no entanglement shared between Alice and Bob, there is no EPR steering effect. □

With straightforward calculation, the maximal value $F^{{}^{\left(n,k\right)}}\left(W\right)$ in the quantum region can be achieved by using the GHZ state $|\psi_{n}^{\theta }\rangle$. That is,

(21) $$F^{{}^{\left(n,k\right)}}\left(W\right)\leq \max_{W}\;F^{{}^{\left(n,k\right)}}\left(W\right)=\;F^{{}^{\left(n,k\right)}}\left(\psi_{n}^{\theta }\right)=R_{n}.$$

By regarding the averages of observables Bell operators as two axes of a plane, we depict the geometrical meaning of (21) in Figure 1. On this basis, we quantitatively characterize the measure of steering for a given state *W* [46] as

$$S\left(W\right)=\left\{0,\frac{F^{\left(n,k\right)}\left(W\right)-R_{n-k}}{R_{n}-R_{n-k}}\right\}.$$

It is easy to verify that (i) 0≤S(W)≤1, (ii) S(W)=0 if these *k* qubits at Alice’s hand cannot steer the state of Bob’s qubits, and (iii) S(W)=1 if the fully-entangled state $|\psi_{n}^{\theta }\rangle$ is initially prepared. As a result, the steering criteria can be geometrically depicted in terms of the expectation values of Bell operators. An alternative geometric extension of the Clauser–Horne inequality for three subsystems is studied by Dutta et al. [47]. Therein, the three-qubit Bell-type and Mermin inequalities are derived by introducing statistical separation of probabilities [47].

To reveal the connection between the steering effect and Bell nonlocality, let us define a *p*-task function

$$F_{p}^{{}^{\left(n,k\right)}}=\sqrt[{p}]{{\left|\langle B_{k}^{+}B_{n-k}\rangle \right|}^{p}+{\left|\langle B_{k}^{+}B_{n-k}^{\prime }\rangle \right|}^{p}}+\sqrt[{p}]{{\left|\langle B_{k}^{-}B_{n-k}\rangle \right|}^{p}+{\left|\langle B_{k}^{-}B_{n-k}^{\prime }\rangle \right|}^{p}}.$$

In the LHV–LHS model, since $\left|{\langle B_{k}^{+}\rangle }_{LHV}\right|,$$\left|{\langle B_{k}^{-}\rangle }_{LHV}\right|\leq 2$ we have

(22) $$F_{\infty }^{{}^{\left(n,k\right)}}\leq \max_{\rho_{\lambda }}\left\{\left|\langle B_{n-k}\rangle \right|,\left|\langle B_{n-k}^{\prime }\rangle \right|\right\}=R_{n}.$$

On the other hand, $F_{\infty }^{{}^{\left(n,k\right)}}=\max\left\{\left|\langle B_{n-k^{\prime }}\rangle \right|,\left|\langle B_{n-k^{\prime }}^{\prime }\rangle \right|\right\}$ if Alice initially prepares the ($n-k^{\prime }$)-qubit GHZ state and then sends (n−k) of them to Bob ($k^{\prime }<k$). Therefore, the violation of (22) indicates that nonlocality distributed among more than (n−k) qubits can be achieved using the EPR-steering.

As the end of the section, we compare $F_{p}^{{}^{\left(n,k\right)}}$ with another *p*-task function

$$T_{p}^{{}^{\left(n,k\right)}}={\left({\left|\langle B_{n}\rangle \right|}^{p}+{\left|\langle B_{n}^{\prime }\rangle \right|}^{p}\right)}^{\frac{1}{p}}.$$

Some remarks are made in order. Firstly, $T_{2}^{{}^{\left(n,k\right)}}$ can be used for the entanglement classification [28,29], and $T_{2}^{{}^{\left(n,k\right)}}\leq F_{2}^{{}^{\left(n,k\right)}}$ as geometrically depicted in Figure 1. Secondly, in the LHV–LHS model, one can verify that $T_{2}^{{}^{\left(n,k\right)}}\leq \max_{\rho_{\lambda }}\sqrt{{\langle B_{n-k}\rangle }_{\rho_{\lambda }}^{2}+{\langle B_{n-k}^{\prime }\rangle }_{\rho_{\lambda }}^{2}}$ and $T_{\infty }^{{}^{\left(n,k\right)}}\leq \max_{\rho_{\lambda }}\left\{\left|\langle B_{n-k}\rangle \right|,\left|\langle B_{n-k}^{\prime }\rangle \right|\right\}$ similar to (14) and (22), respectively. Thirdly, with straightforward calculation and numerical calculation, we have

(23) $$F_{p}^{{}^{\left(n,k\right)}}\left(\psi_{n}^{\theta }\right)=T_{p}^{{}^{\left(n,k\right)}}\left(\psi_{n}^{\theta }\right)=2^{\frac{n}{2}-1}{\left\{{\left|\cos\theta +\sin\theta \right|}^{p}+{\left|\cos\theta -\sin\theta \right|}^{p}\right\}}^{\frac{1}{p}},$$

and

(24) $$\max_{\theta }F_{p\geq 2}^{{}^{\left(n,k\right)}}\left(\psi_{n}^{\theta }\right)=F_{p\geq 2}^{{}^{\left(n,k\right)}}\left(\psi_{n}^{0}\right)=R_{n},$$

which are independent of *k* and *p*. Finally, numerical simulation shows that $\max_{W}F_{p}^{{}^{\left(n,k\right)}}\left(W\right)=R_{n}$∀p≥2.

## 4. Steering Criteria for Qutrit Systems

Inspired by the usefulness of recursive Bell operators for multi-qubit steering, we explore the possibility of tackling quantum steering in a qutrit case. Recently, multi-qutrit Mermin inequalities were proposed by Lawrence [48]. The Bell operators therein are

$$M_{n}^{\left(l\right)}=\frac{1}{3}\left[\prod_{j=1}^{n}⊗\left({\hat{X}}_{j}+\alpha^{2}{\hat{Y}}_{j}\right)+\prod_{j=1}^{n}⊗\omega^{2l}\left({\hat{X}}_{j}+\omega \alpha^{2}{\hat{Y}}_{j}\right)+\prod_{j=1}^{n}⊗\omega^{l}\left({\hat{X}}_{j}+\omega^{2}\alpha^{2}{\hat{Y}}_{j}\right)\right],$$

where l=0,1,2, α=exp(2πi/9),ω=exp(2πi/3). In addition, the Pauli matrix for the qutrit *j* are ${\hat{X}}_{j}=\sum_{n=0}^{2}|n+1\rangle \langle n|$ and ${\hat{Y}}_{j}=\sum_{n=0}^{2}\alpha^{1-3\delta_{n,2}}$$|n+1\rangle \langle n|$ ($\delta_{n,2}=1$ if n=2; 0 otherwise). Here, the hat is for single qutrit operators to distinguish them from qubit ones. Notably, the measurement outcomes can be 1,ω, and $\omega^{2}$. One can revise $M_{n}^{\left(l\right)}$ as the recursive forms

$$\begin{array}{cc} M_{n}^{\left(0\right)} & =M_{k}^{\left(0\right)}M_{n-k}^{\left(0\right)}+M_{k}^{\left(1\right)}M_{n-k}^{\left(2\right)}+M_{k}^{\left(2\right)}M_{n-k}^{\left(1\right)},\\ M_{n}^{\left(1\right)} & =M_{k}^{\left(0\right)}M_{n-k}^{\left(1\right)}+M_{k}^{\left(1\right)}M_{n-k}^{\left(0\right)}+M_{k}^{\left(2\right)}M_{n-k}^{\left(2\right)},\\ M_{n}^{\left(2\right)} & =M_{k}^{\left(0\right)}M_{n-k}^{\left(2\right)}+M_{k}^{\left(1\right)}M_{n-k}^{\left(1\right)}+M_{k}^{\left(2\right)}M_{n-k}^{\left(0\right)}. \end{array}$$

Similarly to the qubit case, we define a *p*-task function as

$$R_{p}^{\left(n,k\right)}=\frac{1}{3}\sum_{j=0}^{2}{\left|\sum_{l=0}^{2}{\langle M_{k}^{\left(j\right)}M_{n-k}^{\left(l\right)}\rangle }^{p}\right|}^{\frac{1}{p}}.$$

On the other hand, let

$$A_{m}^{\left(j\right)}=\sum_{l=0}^{2}\omega^{lj}M_{m}^{\left(l\right)},\;M_{m}^{\left(l\right)}=\sum_{l=0}^{2}\omega^{-lj}A_{m}^{\left(j\right)},$$

and we define an alternative *p*-task function

$$L_{p}^{\left(n,k\right)}=\frac{1}{3}\sum_{j=0}^{2}{\left|\sum_{l=0}^{2}{\langle A_{k}^{\left(j\right)}M_{n-k}^{\left(l\right)}\rangle }^{p}\right|}^{\frac{1}{p}}.$$

As for the LHV–LHS model, it is sufficient that for an unsteerable *W*,

(25) $$L_{p}^{\left(n,k\right)}\left(W\right)\leq A_{LHV(k)}^{\max}M_{n-k,p}^{\max},\;\mathrm{and}\;R_{p}^{\left(n,k\right)}\left(W\right)\leq M_{LHV(k)}^{\max}M_{n-k,\;p}^{\max},$$

where $A_{LHV(k)}^{\max}=\frac{1}{3}\max_{\lambda }\sum_{j=0}^{2}\left|{\langle A_{k}^{\left(j\right)}\rangle }_{LHV}\right|,$ $M_{LHV(k)}^{\max}=\frac{1}{3}\max_{\lambda }\sum_{j=0}^{2}\left|{\langle M_{k}^{\left(j\right)}\rangle }_{LHV}\right|$, and $M_{n-k,\;p}^{\max}=\max_{\rho_{\lambda }}{\left|\sum_{l=0}^{2}{\langle M_{n-k}^{\left(l\right)}\rangle }_{\rho_{\lambda }}^{p}\right|}^{\frac{1}{p}}$. For lower values of *n*, the values of the both hands sides for p=2 are numerically evaluated in Table 1 and Table 2. The numerical simulation shows that $A_{LHV(k)}^{\max}$ and $M_{LHV(k)}^{\max}$ can be achieved using the LHV with the uniform outcome,

$$\lambda_{\mathrm{uniform}}=\left\{\left({\hat{M}}_{j}^{i_{j}},o_{j}^{i_{j}}\right)|o_{j}^{i_{j}}\left({\hat{M}}_{j}^{i_{j}}\right)=x,\;\forall {\hat{M}}_{j}^{i_{j}}\in \left\{{\hat{X}}_{j},{\hat{Y}}_{j}\right\}\right\},$$

where $x\in \{1,\omega ,\omega^{2}\}$. Given p=2, however, it is difficult to find $M_{n-k,\;2}^{\max}$ since it may concern the entanglement classification in the three-level case, which needs further exploration that is beyond our scope. In addition, since the connection between joint measurability and unsteerability is also unclear for qutrits, one cannot determine whether $L_{2}$ outperforms $R_{2}$ to serve as steering criteria. Finally, as *p* goes infinity, the steering inequalities become

(26) $$L_{\infty }^{\left(n,k\right)}\leq A_{LHV(k)}^{\max}T_{Q}^{\left(n,k\right)},\;R_{\infty }^{\left(n,k\right)}\leq M_{LHV(k)}^{\max}T_{Q}^{\left(n,k\right)},$$

where $T_{Q}^{\left(n,k\right)}=\max_{\left\{l,m\right\}}\left\{|\langle \psi_{m}^{\left(n-k\right)}|M_{n-k}^{\left(l\right)}|\psi_{m}^{\left(n-k\right)}\rangle |\right\}$ and $|\psi_{m}^{\left(n-k\right)}\rangle$ represents the GHZ state for (n−k) qutrits given by Lawrence [48]. Therefore, the violation of (26) indicates that nonlocality distributed among more than (n−k) qutrits can be achieved using the EPR-steering.

## 5. Steering Criteria of the Star-Shaped Quantum Network

We apply the result to test the steering effect in a star-shaped quantum network [49]. Therein, as the center of the star network, Alice initially prepares the $W_{\left(l\right)}$ of $n_{\left(l\right)}$ subsystems, and then sends the $\left(n_{\left(l\right)}-k_{\left(l\right)}\right)$ subsystems of $W_{\left(l\right)}$ to distant end-user Bob${}_{\left(l\right)}$, where l=1, 2,..., *L*. Similarly, Bob${}_{\left(l\right)}$ measures the observable $M_{j^{\prime },\left(l\right)}^{0}$ or $M_{j^{\prime },\left(l\right)}^{1}$ on the $j^{\prime }$-th qubit at hand $\left(k_{\left(l\right)}+1\leq j^{\prime }\leq n_{\left(l\right)}\right)$, and Alice sends Bob${}_{\left(l\right)}$ the content of the input–output set $c_{\left(l\right)}=\left\{\left(M_{j,(l)}^{i_{j}},o_{j,(l)}^{i_{j}}\right)\right|1\leq j\leq k_{\left(l\right)},$$i_{j}\in \left\{0,1\right\}\}$ via one-way classical communication. Each Bob${}_{\left(l\right)}$ can perform local measurements and then derive the values

(27) $$\sqrt{{\langle B_{n_{\left(l\right)}-k_{\left(l\right)}}\rangle }^{2}+{\langle B_{n_{\left(l\right)}-k_{\left(l\right)}}^{\prime }\rangle }^{2}}=r_{l}\leq R_{n_{\left(l\right)}-k_{\left(l\right)}}.$$

Define the quantities $I_{\pm }=\prod_{l=1}^{L}\frac{1}{r_{l}}\sqrt{{\langle B_{k_{\left(l\right)}}^{\pm }B_{n_{\left(l\right)}-k_{\left(l\right)}}\rangle }^{2}+{\langle B_{k_{\left(l\right)}}^{\pm }B_{n_{\left(l\right)}-k_{\left(l\right)}}^{\prime }\rangle }^{2}}$. For any LHV–LHS model, we have

$$\begin{array}{cc} \; & {\left|I_{+}\right|}^{\frac{1}{L}}+{\left|I_{-}\right|}^{\frac{1}{L}}\\ \; & =\sum_{\omega =+,-}{\left\{\prod_{l=1}^{L}\frac{\left|{\langle B_{k_{\left(l\right)}}^{\omega }\rangle }_{LHV}\right|}{r_{l}}\sqrt{{\langle B_{n_{\left(l\right)}-k_{\left(l\right)}}\rangle }_{LHS}^{2}+{\langle B_{n_{\left(l\right)}-k_{\left(l\right)}}^{\prime }\rangle }_{LHS}^{2}}\right\}}^{\frac{1}{L}}\\ \; & =\sum_{\omega =+,-}{\left\{\prod_{l=1}^{L}\left|{\langle B_{k_{\left(l\right)}}^{\omega }\rangle }_{LHV}\right|\right\}}^{\frac{1}{L}}\\ \; & \leq \prod_{l=1}^{L}{\left\{\left|{\langle B_{k_{\left(l\right)}}^{+}\rangle }_{LHV}\right|+\left|{\langle B_{k_{\left(l\right)}}^{-}\rangle }_{LHV}\right|\right\}}^{\frac{1}{L}}\\ \; & =\prod_{l=1}^{L}{\left\{\left|\frac{{\langle B_{k_{\left(l\right)}}\rangle }_{LHV}+{\langle B_{k_{\left(l\right)}}^{\prime }\rangle }_{LHV}}{2}\right|+\left|\frac{{\langle B_{k_{\left(l\right)}}\rangle }_{LHV}-{\langle B_{k_{\left(l\right)}}^{\prime }\rangle }_{LHV}}{2}\right|\right\}}^{\frac{1}{L}}\\ \; & \leq \prod_{l=1}^{L}{\left\{\max\left(\left|{\langle B_{k_{\left(l\right)}}\rangle }_{LHV}\right|,\left|{\langle B_{k_{\left(l\right)}}^{\prime }\rangle }_{LHV}\right|\right)\right\}}^{\frac{1}{L}}\leq 1, \end{array}$$

where the first inequality is as a result of Mahler’s inequality [50]. On the other hand, let $W_{\left(l\right)}$ be an $n_{\left(l\right)}$-qubit quantum state for all *l*. We have

$$\begin{array}{cc} \; & \max\left({\left|I_{+}\right|}^{\frac{1}{L}}+{\left|I_{-}\right|}^{\frac{1}{L}}\right)\\ \; & =\sum_{\omega =+,-}{\left\{\prod_{l=1}^{L}\frac{1}{r_{l}}\sqrt{{\langle B_{k_{\left(l\right)}}^{\omega }B_{n_{\left(l\right)}-k_{\left(l\right)}}\rangle }^{2}+{\langle B_{k_{\left(l\right)}}^{\omega }B_{n_{\left(l\right)}-k_{\left(l\right)}}^{\prime }\rangle }^{2}}\right\}}^{\frac{1}{L}}\\ \; & \leq {\left\{\prod_{l=1}^{L}\sum_{\omega =+,-}\frac{1}{r_{l}}\sqrt{{\langle B_{k_{\left(l\right)}}^{\omega }B_{n_{\left(l\right)}-k_{\left(l\right)}}\rangle }^{2}+{\langle B_{k_{\left(l\right)}}^{\omega }B_{n_{\left(l\right)}-k_{\left(l\right)}}^{\prime }\rangle }^{2}}\right\}}^{\frac{1}{L}}\\ \; & =\prod_{l=1}^{L}{\left\{\sum_{\omega =+,-}\frac{F^{\left(n_{\left(l\right)},k_{\left(l\right)}\right)}}{r_{l}}\right\}}^{\frac{1}{L}}\leq \prod_{l=1}^{L}{\left\{\frac{R_{n_{\left(l\right)}}}{r_{l}}\right\}}^{\frac{1}{L}}, \end{array}$$

where, according to Mahler’s inequality, the equality of the first inequality holds if the ratio

(28) $$\frac{\sqrt{{\langle B_{k_{\left(l\right)}}^{-}B_{n_{\left(l\right)}-k_{\left(l\right)}}\rangle }^{2}+{\langle B_{k_{\left(l\right)}}^{-}B_{n_{\left(l\right)}-k_{\left(l\right)}}^{\prime }\rangle }^{2}}}{\sqrt{{\langle B_{k_{\left(l\right)}}^{+}B_{n_{\left(l\right)}-k_{\left(l\right)}}\rangle }^{2}+{\langle B_{k_{\left(l\right)}}^{+}B_{n_{\left(l\right)}-k_{\left(l\right)}}^{\prime }\rangle }^{2}}}=C\;\;\forall l.$$

In addition, if the entanglement index of $n_{\left(l\right)}$ qubits are larger than that of $n_{\left(l\right)}-k_{\left(l\right)}$ qubits ($E_{n_{\left(l\right)}}>E_{n_{\left(l\right)}-k_{\left(l\right)}}$), we have

(29) $$1<\frac{R_{n_{\left(l\right)}}}{R_{n_{\left(l\right)}-k_{\left(l\right)}}}\leq \frac{R_{n_{\left(l\right)}}}{r_{l}}.$$

As a result, if the network cannot be simulated using LHV–LHS models, we have $\max({\left|I_{+}\right|}^{\frac{1}{L}}+{\left|I_{-}\right|}^{\frac{1}{L}})>1$. For example, let the state $W_{\left(l\right)}$ be $n_{\left(l\right)}$-qubit GHZ state, then we have $E_{n_{\left(l\right)}}=n_{\left(l\right)}\geq 3$ and $E_{n_{\left(l\right)}-k_{\left(l\right)}}=2$. In addition, let (28) hold and $r_{l}=R_{n_{\left(l\right)}-k_{\left(l\right)}}=\sqrt{2}$ ($E_{n_{\left(l\right)}-k_{\left(l\right)}}=2$). As a result, we have $\frac{R_{n_{\left(l\right)}}}{r_{l}}=\sqrt{2^{n_{\left(l\right)}-2}}\geq \sqrt{2}>1$, and hence

$$\max\left({\left|I_{+}\right|}^{\frac{1}{L}}+{\left|I_{-}\right|}^{\frac{1}{L}}\right)={\left(\prod_{l=1}^{L}\sqrt{2^{n_{\left(l\right)}-2}}\right)}^{\frac{1}{L}}\geq 2^{\frac{1}{2}}>1.$$

The steering effect in the quantum networks can be corrupted by the detection efficiency, the noise in the state, the misalignment of measurement settings, and the loss. Although the full discussion on the real-world limitations is beyond our scope, we can consider the unbiased noise in the state as a simple example. Let the density matrix of the contaminated state $W_{\left(l\right)}$ be

$$\rho_{\left(l\right)}=p|\psi_{n_{\left(l\right)}}^{\theta =0}\rangle \langle \psi_{n_{\left(l\right)}}^{\theta =0}|+\left(1-p\right)I_{\left(l\right)}\;\;\forall l,$$

where $I_{\left(l\right)}$ denotes the $2^{n_{\left(l\right)}}\times 2^{n_{\left(l\right)}}$ identity matrix. It is easy to show that, if $p{\left(\prod_{l=1}^{L}\sqrt{2^{n_{\left(l\right)}-2}}\right)}^{\frac{1}{L}}\leq 1$, we have

$$\max\left({\left|I_{+}\right|}^{\frac{1}{L}}+{\left|I_{-}\right|}^{\frac{1}{L}}\right)=p{\left(\prod_{l=1}^{L}\sqrt{2^{n_{\left(l\right)}-2}}\right)}^{\frac{1}{L}}\leq 1,$$

which indicates the vanishing of the steering effect.

There is an evident advantage for the experimental realization of these proposed steering inequalities. Since these steering inequalities are proposed in terms of Bell operators, the experiment realization of steering inequalities are exactly the same as the Bell-type experiments involving these Bell operators. In this case, the experimental input-output data of either multi-qubit Bell–Klyshko or multi-qutrit Mermin–Lawrence inequalities can be exploited for testing both Bell nonlocality and quantum (un)steerability.

Finally, it is interesting to test EPR steering in the optical way. For example, the tripartite EPR steering has been discussed by using a three-mode Gaussian state created by four-wave mixing in Rubidium atoms using linear and nonlinear beam splitters [51]. Notably, the four-wave mixing process can be generated using twin beams [52], and can be employed in intensity-difference squeezing via energy-level modulations in high-gain atomic media [53]. The related experiments to realize multi-mode noise correlation in an atomic ensemble or an atomic-like medium have been developed in [52,53].

## 6. Conclusions

In conclusion, we demonstrate the usefulness of the two-task function comprising the superposition of the Bell operators. These two-task functions can be employed to detect steerability in the bipartite multi-qubits/qutrits scenario, and reveal the connection between joint measurability and quantum unsteerability. On the other hand, we propose the geometrical measure in terms of a two-task function. In this way, we shed light on deriving EPR steering inequalities by using the connection between EPR steering, entanglement, and Bell nonlocality. Furthermore, such task functions can be further exploited in detecting steerability in the star-shaped quantum networks. Finally, it is interesting to further explore EPR steering in the multi-level case, where the multilevel entanglement classification should play an essential role but relatively little is known about it.

## Figures and Tables

**Table 1:** The achievable maximal values of $L_{2}^{\left(n,k\right)}$ (with respect to the GHZ states) and $A_{LHV(k)}^{\max}M_{n-k,2}^{\max}$ for n=3,4,5,6 and k=1,2,⋯,n−1. Each entry represents the numerical values of $L_{2}^{\left(n,k\right)}/A_{LHV(k)}^{\max}M_{n-k,2}^{\max}$.

n\k	1	2	3	4	5
3	1.88243/1.61752	1.85716/1.26589			
4	2.97211/2.28049	3.73907/2.58199	3.32009/2.16811		
5	4.85581/4.55593	4.89475/3.64027	5.77006/4.42222	6.37854/3.79766	
6	9.1105/8.07617	9.35794/7.27247	8.92001/6.23477	11.5972/7.74597	12.4478/6.72884

**Table 2:** The achievable maximal values of $R_{2}^{\left(n,k\right)}$ (with respect to the GHZ states) and $M_{LHV(k)}^{\max}M_{n-k,2}^{\max}$ for n=3,4,5,6 and k=1,2,⋯,n−1. Each entry represents the numerical values of $R_{2}^{\left(n,k\right)}/M_{LHV(k)}^{\max}M_{n-k,2}^{\max}$.

n\k	1	2	3	4	5
3	0.880746/0.860663	1.08866/0.843924			
4	1.44016/1.21342	1.76149/1.72133	2.17732/1.47687		
5	2.18574/2.42416	2.63165/2.42685	3.18657/3.01232	4.35465/2.78729	
6	4.58661/4.29724	4.37148/4.84832	5.2633/4.24699	6.37314/5.68515	8.7093/5.08017
